# Improved cookstoves in low-resource settings: a spur to successful implementation strategies

**DOI:** 10.1038/s41533-019-0148-4

**Published:** 2019-10-11

**Authors:** M. Thakur, C. P. van Schayck, E. A. Boudewijns

**Affiliations:** Department of Family Medicine, Care and Public Health Research Institute (CAPHRI), Maastricht University, P.O. Box 616, 6200 MD Maastricht, the Netherlands

**Keywords:** Public health, Respiratory tract diseases

Van Gemert et al. have very well described the effectiveness and implementation of improved cookstoves (ICS)/heaters in low- and middle-income countries (LMICs).^1^ Significant reductions in particulate matter (PM_2.5_) and carbon monoxide (CO) concentrations, respiratory symptoms, chest infections, and school absence were found. The results in terms of PM_2.5_, CO, and health benefits were considerable, and therefore in line with previous studies.^2–4^

Over the past few decades, various initiatives have been attempting to implement ICS to reduce household air pollution (HAP) and fuel use, and to improve health conditions.^5,6^ Although positive outcomes linked to ICS have been demonstrated, and clean stoves and fuels are available, the uptake and consistent use has been low.^7^ We will shortly discuss a few key points that might foster successful implementation, including community-focused approaches, the creation of public awareness, usage information, the assurance of maintenance, the involvement of women and an appropriate business model.^8^

Adoption of a new technology does not usually happen overnight; it is a process influenced by a range of personal and surrounding factors.^5,9^ Literature suggests that availability and affordability of technology is not enough, but that understanding of the cultural traditions, especially cooking traditions, and social–economic factors are vital for successful implementation.^4,5,9–14^ Consequently, the ICS should be designed in such a way that it is compatible with social expectations and consistent with local needs and culture. The situational analysis in the study of Van Gemert et al. is therefore a recommendable strategy.^1^

Studies have demonstrated low awareness and knowledge of health risks concerning traditional cookstoves.^15,16^ Creating public awareness might increase uptake and potentially sustained use.^14,16^ The important points to consider are identification of target groups, assessment of existing knowledge and perceptions regarding HAP and health, the kind of information that needs to be communicated and a pragmatic approach to communicate this information, for example via posters, media or local cooking demonstrations.^8^ One of the overlooked aspects in the implementation of ICS is the influence of social network.^17^ Limited studies have attempted to explore this dimension and have found that word-of-mouth among women is a primary persuasive method to adopt ICS.^12,14^ Based on experience of a randomised controlled trial, conducted in urban slums in Bangalore, we experienced that if some women were not happy with the stove, they would influence their neighbours who in turn would refuse to even give it a try.^18^ To resolve this problem, we came up with an educational film featuring some women from similar communities who were enthusiastic about using the ICS and understood its benefits in terms of health very well. Social network analysis could be interesting and beneficial to explore when it comes to implementation of ICS.

Not only awareness needs to be created, but users also need to be informed about the correct use of the ICS. Any progress will only be made if women are using the stove in the correct manner.^8^ Van Gemert et al. demonstrated the use of the ICS before distribution, which might increase correct use of the stoves.^1^ During our study in Indian slums, we experienced that, although we developed the stove in co-operation with the local community, people will not always correctly use the stove.^18^ For example, they will remove the top part of the stove. To inform the users about the correct use of the ICS, we developed a poster for each slum house.

The sustainability of ICS programmes has been, and still is, under debate. Apart from factors mentioned earlier such as affordability and compatibility with cultural habits, this might be due to lack of maintenance and repair/replacement of ICS at the community level.^10,14,19^ Several studies have demonstrated that, after various years, stoves were not used anymore due to damage, and consequently people were going back to using the traditional stove.^10,20^ It is therefore recommendable to ensure maintenance, repair and replacement of the ICS after implementation.^20^ Women can effectively contribute to the distribution and repair networks because of their rapport with the local communities.^14^

Transport and hassle-free repair or replacement can be facilitated by a decentralised production. This would also open opportunities for earning livelihoods, especially for women. Women being the primary users drive demand for the ICS and therefore should be actively involved in the entire value chain, right from constructing and selling to installing and after-sale services.^21,22^ This untapped resource can spur the scale-up of cookstove programmes.^21,22^ Involving women in the entire chain might yield positive benefits for themselves and their families.^22^ Enhancing women’s business skills should be considered, to strengthen their confidence in running their own small business.^8^

Access to financial resources is a key factor in ICS programmes, as both the supply and demand side need the required capital to invest.^8^ Major funders of ICS have emphasised that a market-based approach is a prerequisite for a sustainable and scalable ICS programme.^23^ Market-based approaches are seen as the most effective ways to allocate resources. There are several advantages of market-based approaches, including the lowering of prices and the increase in quality due to competition, the effective delivery of services—and related the exclusion of corruption from highly centralised polities, the increase in uptake because consumers are paying for the stoves themselves and the independency of subsidies or funding.^23^ However, while we agree that organisations should be as independent as possible, a fully commercialised approach presents challenges for organisations who work in low-resource settings.^23,24^ ICS are challenging products to sell, because among others, the market is not demand driven.^24^ Besides, the investment in research and development, the promotion to adopt an ICS, monitoring and evaluation, the assurance of quality and the enabling of credit-based sales are challenging for small local businesses that disseminate ICS.^23^ Regarding the latter, the price of the cookstove is often too expensive for the poor to buy in one time, while it is too low to qualify for bank loans.^24^ Consequently, lower-income households are penalised by the pursuit of fully commercialised approaches. One solution is to simplify the stove, and therefore bring down the costs.^8^ Innovative business models like microfinance might also be considered.^8^ To avoid business costs associated with developing microfinance infrastructure, it is recommendable to work with existing microfinance institutions that are willing to provide a long-term loan for lower interest rates.^24^ Ideally, the repayment rates of these loans are generated through fuel savings.^8^ Carbon financing can be another alternative in the global market for clean cookstoves.^14,25^ While organisations should be commercialised as much as possible, additional (start-up) finance might be needed to also reach the poorest, especially because public health is at stake. ICS therefore require innovative business models.^24^

To conclude, ICS have shown to be effective in reducing HAP and improving health in research settings, but implementation of the stoves on a large scale has been challenging. It is remarkable that only few studies have assessed best ways of disseminating stoves. We argue that good implementation strategies embark on context evaluations—identifying the needs and habits of the target groups—and co-creating ICS. This means that there is no one-size-fits-all approach. Furthermore, public awareness needs to be created, demonstrations about correct use of the ICS should be given and maintenance should be assured. Leveraging women’s strengths can offer an opportunity for the dissemination of ICS. A business model with a balance between commercialisation and (start-up) state/donor support needs to be set up. An integrated approach involving different stakeholders and a cohesive vision would aid in successful implementation of ICS. Further research is imperative to gain insight into the pros and cons of different implementation strategies in diverse contexts. We urgently need to make progress in improving implementation strategies for ICS, as we can gain immense health improvements, reduce environmental impacts and create job opportunities for, especially, poor women. To fight the killers to which almost 3 billion people are exposed to, implementation of ICS should be a top priority.

## References

[1] van Gemert F (2019). Effects and acceptability of implementing improved cookstoves and heaters to reduce household air pollution: a FRESH AIR study. NPJ Prim. Care Respir. Med..

[2] Thakur M (2018). Impact of improved cookstoves on women’s and child health in low and middle income countries: a systematic review and meta-analysis. Thorax.

[3] Quansah R (2017). Effectiveness of interventions to reduce household air pollution and/or improve health in homes using solid fuel in low-and-middle income countries: a systematic review and meta-analysis. Environ. Int..

[4] Thomas E, Wickramasinghe K, Mendis S, Roberts N, Foster C (2015). Improved stove interventions to reduce household air pollution in low and middle income countries: a descriptive systematic review. BMC Public Health.

[5] Urmee T, Gyamfi S (2014). A review of improved cookstove technologies and programs. Renew. Sust. Energ. Rev..

[6] Vahlne N, Ahlgren EO (2014). Policy implications for improved cook stove programs—a case study of the importance of village fuel use variations. Energ. Policy.

[7] Hollada J (2017). Perceptions of improved biomass and liquefied petroleum gas stoves in Puno, Peru: implications for promoting sustained and exclusive adoption of clean cooking technologies. Int. J. Environ. Res. Public Health.

[8] Deutsche Gesellschaft für Internationale Zusammenarbeit (GIZ). *Cooking Energy Compendium. A Practical Guidebook for Implementers of Cooking Energy Interventions* (2013).

[9] Ruiz-Mercado I, Masera O, Zamora H, Smith KR (2011). Adoption and sustained use of improved cookstoves. Energ. Policy.

[10] Wolf J, Mäusezahl D, Verastegui H, Hartinger S (2017). Adoption of clean cookstoves after improved solid fuel stove programme exposure: a cross-sectional study in three Peruvian Andean regions. Int. J. Environ. Res. Public Health.

[11] Donegan, J. *Design and Implementation of a Ferrocement Improved Cookstove in Rural Panama* (2018).

[12] Vigolo V, Sallaku R, Testa F (2018). Drivers and barriers to clean cooking: A systematic literature review from a consumer behavior perspective. Sustainability.

[13] Checkley W (2016). Managing threats to respiratory health in urban slums. Lancet Resp. Med..

[14] Global Alliance for Clean Cookstoves. *Igniting Change: A Strategy for Universal Adoption of Clean Cookstoves and Fuels* (2011).

[15] Ghergu C (2016). Dealing with indoor air pollution: an ethnographic tale from urban slums in Bangalore. Int. J. Health S. Res..

[16] Alam A (2016). Household air pollution intervention implications: findings from qualitative studies and a field trial of clean cookstoves in two rural villages in India. Int. J. Environ. Res. Public Health.

[17] Vulturius, G. & Wanjiru, H. Role of social relations in the adoption of improved cookstoves. (Stockholm Environment Institute, 2017).

[18] Thakur M (2017). Low-smoke chulha in Indian slums: study protocol for a randomised controlled trial. BMC Public Health.

[19] Lim J, Petersen S, Schwarz D, Schwarz R, Maru D (2013). A rights-based approach to indoor air pollution. Health Hum. Rights.

[20] Schilmann A (2019). A follow-up study after an improved cookstove intervention in rural Mexico: estimation of household energy use and chronic PM2.5 exposure. Environ. Int..

[21] Global Alliance for Clean Cookstoves. *Scaling Adoption of Clean Cooking Solutions through Women’s Empowerment* (2013).

[22] Shankar A (2014). Maximizing the benefits of improved cookstoves: moving from acquisition to correct and consistent use. Glob. J. Health Sci..

[23] Bailis R, Cowan A, Berrueta V, Masera O (2009). Arresting the killer in the kitchen: the promises and pitfalls of commercializing improved cookstoves. World Dev..

[24] Selco Foundation. Improved cook stoves: lessons learnt.

[25] Lambe, F., Jürisoo, M., Lee, C. & Johnson, O. Can carbon revenues help transform household energy markets? A scoping study with cookstove programmes in India and Kenya. (Stockholm Environment Institute, 2014).

